# 689. *Streptococcus suis* Endocarditis: Echocardiographic Features and Clinical Outcomes

**DOI:** 10.1093/ofid/ofab466.886

**Published:** 2021-12-04

**Authors:** Kornkanok Trirattanapa, Quanhathai Kaewpoowat, Rungsrit Kanjanavanit

**Affiliations:** Faculty of Medicine, Chiang Mai University, Thailand, chiang mai, Chiang Mai, Thailand

## Abstract

**Background:**

*Streptococcus suis (S. suis*) is a zoonotic pathogen that transmits to the human with direct contact of pig or raw pork ingestion. This infection has been described in Asia, especially Thailand, Vietnam, and China. *S. suis* could cause wide range of infection, including endocarditis. This study aimed to describe the clinical features, echocardiogram findings, and outcomes of *S. suis* endocarditis.

**Methods:**

A single center, ten-year (January 2009 to December 2018), retrospective cohort was conducted among patients who were diagnosed with *S.suis* endocarditis in 1,200-bed hospital in Northern, Thailand.

**Results:**

Forty-three patients of *S.suis* endocarditis were identified during the study period. Of those, 28 (65%) patients had positive blood culture and 15 (35%) was diagnosed by 16SRNA bacterial identification from heart valve tissue. Majority (81%) were male with median age of 35. There were 62 affected valves in 43 patients. Twenty patients (48%) had vegetation larger than 10 mm in diameter and 35 (81.4%) patients had moderately severe or severe valvular regurgitation. Valvular perforation was described in 23 patients (53%). Perivalvular complications were founded in 15 patients (35%). Systemic embolism occurred in 17 (40%) patients. Cardiac operation was undertaken in 35 (81%) patients. There were 2 in-hospital deaths (5%) and 6 patients (14%) had disabilities. Moderately severe/severe regurgitation, systemic embolism, and no cardiac operation were significantly associated with disability or death from univariate analysis. By logistic regression analysis, systemic embolism was the only risk factor for disability or death (OR = 12.6, 95% CI 1.3-123.5, p = 0.029).

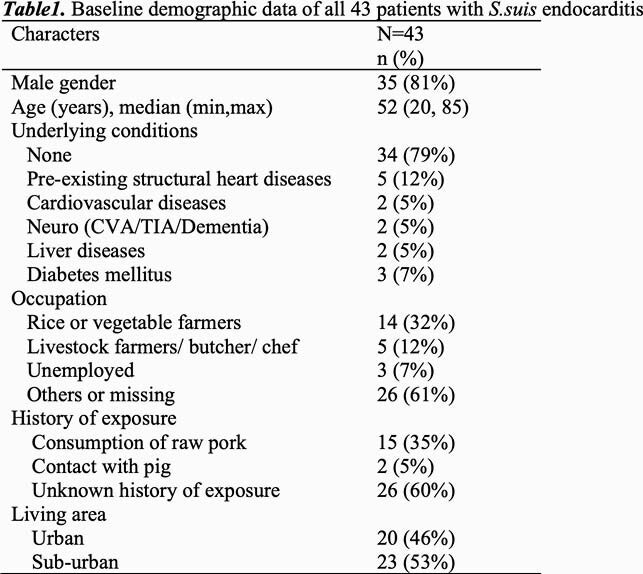

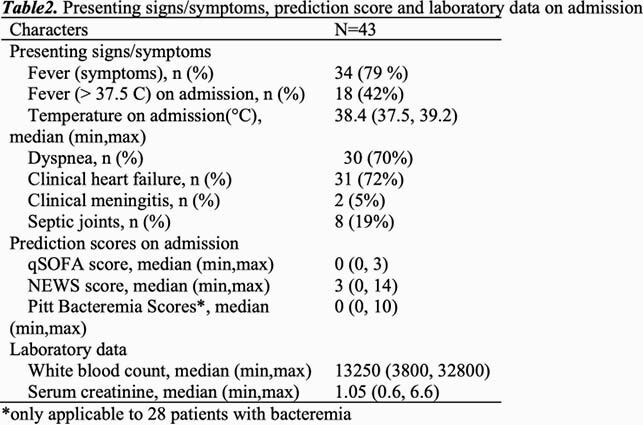

Presenting signs/symptoms, prediction score and laboratory data on admission

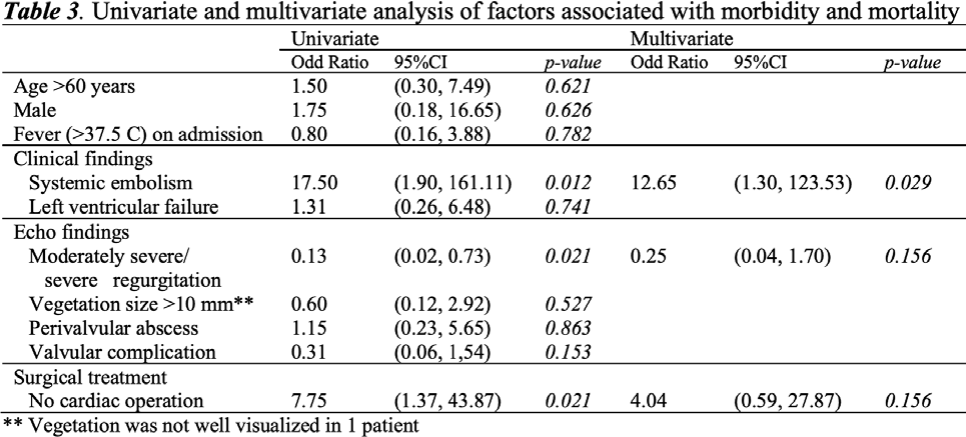

**Conclusion:**

*S. suis* endocarditis had high rate of valvular damage with complications and resulting systemic embolism. Surgery is required in majority of the patients. Embolism was associated with disability or death.

**Disclosures:**

**All Authors**: No reported disclosures

